# Implementation science in nutrition: a summary and synthesis

**DOI:** 10.1017/S1368980021000884

**Published:** 2021-04

**Authors:** Haribondhu Sarma

**Affiliations:** 1 Research School of Population Health, Colleague of Health and Medicine, The Australian National University, Canberra, ACT 2601, Australia; 2 Nutrition and Clinical Services Division, icddr,b, Dhaka 1212, Bangladesh

**Keywords:** Implementation science, Home fortification, Scaling-up, Cost-effectiveness, Sustainability

## Abstract

**Objective:**

This paper aimed to summarise and critically synthesise the key findings of the articles included in the supplement entitled ‘Nutrition Implementation Science: The Experience of a Large-Scale Home Fortification in Bangladesh’.

**Design:**

Commentary, summary and synthesis.

**Settings:**

Low- and middle-income country.

**Results:**

The supplement included six articles, including this summary paper. The second article presented an implementation science framework that facilitated conceptualising and evaluating the home-fortification programme in Bangladesh implemented by the Bangladesh Rural Advancement Committee (BRAC). The framework encompasses five components: identifying an ‘effective’ intervention; scaling-up and implementation fidelity; course corrections during implementation and assessing the implementation’s effectiveness; promoting sustainability of interventions and consideration of a concurrent evaluation to identify ‘effective’ interventions and to assess the process and outcome indicators of implementation. The other four articles in this supplement addressed the different components of the framework. For example, the third article addressed the implementation fidelity of a home-fortification programme, and the fourth article described the use of concurrent evaluation to course correct the implementation plan that resulted in improved implementation fidelity. The fifth article explained the outcome of course correction in the programme coverage, and the sixth article described the cost-effectiveness of the BRAC home-fortification programme.

**Conclusions:**

Overall, the supplement provides a comprehensive understanding of nutrition implementation science, which is very new in the field. The lessons learned in this supplement may enhance the capacity of researchers, policymakers and key stakeholders in the nutrition field to scale up new nutrition interventions and sustain them until malnutrition is alleviated.

Implementation science has been defined as a systematic study to identify an ‘effective’ intervention for scaling-up in a real-world setting, considering real-time evaluation and a feedback-loop mechanism to improve implementation and effectiveness to sustain the intervention and its outcome over the long term^(1)^. The use of implementation science in nutrition is a recent initiative that is gradually becoming more recognised among nutrition stakeholders, including researchers, implementers and funders. Over the past few decades, several nutrition interventions have been invented, tested and found to be effective in alleviating various forms of malnutrition. However, the contribution of these innovative interventions to overall human development has been minimal. One theory is that this could be improved using implementation science to thoughtfully scale up these interventions. These interventions, initially tested in small-scale and controlled community settings, have proved effective, but they have not been piloted to investigate how they may work in larger-scale, real-world implementation settings. Despite the availability of well-tested approaches to address most forms of malnutrition, the international community and most governments in developing countries have not been as successful as they would like in tackling malnutrition^(2)^. Therefore, a high burden of malnutrition still exists^(3)^ and imposes adverse consequences for health and overall well-being^(4)^. In this context, this supplement aimed to develop and test a comprehensive conceptual framework for implementation science to accelerate the scaling-up of nutrition interventions in real-world settings.

This paper aimed to summarise and critically synthesise the key findings and conclusions of the following five articles included in the supplement entitled ‘Nutrition Implementation Science: The Experience of a Large-Scale Home Fortification in Bangladesh’:

*Paper 2:* Sarma H, D’Este C, Ahmed T *et al.* (2020) Developing a conceptual framework for implementation science to evaluate a nutrition intervention scaled-up in a real-world setting. *Public Health Nutr*. Feb 27:1–6. https://doi.org/10.1017/S1368980019004415.
*Paper 3:* Sarma H, Tariqujjaman M, Mbuya MN *et al.* (2020) Factors associated with home visits by volunteer community health workers to implement a home-fortification intervention in Bangladesh: a multilevel analysis. *Public Health Nutr*. Jan 27:1–4. https://doi.org/10.1017/S1368980019003768.
*Paper 4:* Sarma H, Uddin MF, Islam MA *et al.* (2020) Use of concurrent evaluation to improve implementation of a home fortification programme in Bangladesh: a methodological innovation. *Public Health Nutr*. Mar 5:1–1. https://doi.org/10.1017/S1368980020000439.
*Paper 5:* Sarma H, Mbuya MN, Tariqujjaman M *et al.* (2020) Role of home visits by volunteer community health workers: to improve the coverage of micronutrient powders in rural Bangladesh. *Public Health Nutr*. Mar 5:1–1. https://doi.org/10.1017/S1368980020000038.
*Paper 6:* Ahmed S, Sarma H, Hasan MZ *et al.* (2020) Cost-effectiveness of a Market-Based Home-fortification of Food with Micronutrient Powder Programme in Bangladesh. *Public Health Nutr*. Oct 29:1–2. https://doi.org/10.1017/S1368980020003602.

## Conceptual framework: a holistic understanding of implementation science

This supplement demonstrated the use of a nutrition implementation science framework to facilitate the conceptualisation and understanding of a nutrition intervention that was comprehensively implemented in a real-world setting. The second paper of this supplement proposed a conceptual framework with five components: (1) identifying an ‘effective’ intervention; (2) scaling-up and implementation fidelity; (3) course corrections during implementation; (4) promoting sustainability of interventions and (5) consideration of a comprehensive methodological paradigm^(1)^. The other four articles (Papers 3–6) in this supplement address the different components of the framework. For example, the third article addresses the implementation fidelity of a home-fortification programme^(5)^, and the fourth article describes the use of concurrent evaluation to course correct the implementation plan that resulted in improved implementation fidelity^(6)^. The fifth article explains the outcome of course correction in the programme coverage^(7)^, and the sixth article describes the cost-effectiveness of the Bangladesh Rural Advancement Committee (BRAC) home-fortification programme^(8)^. The following sections of this paper clarify each of the components of our implementation science framework, summarise key findings reported in Papers 3–6 of this supplement and critically synthesises these findings with support from the relevant literature.

## Identifying an ‘effective’ intervention and BRAC home fortification

The first component of the framework helped to identify an ‘effective’ intervention, whereby our framework proposes four intervention characteristics: intervention sources, evidence strength and quality, relative advantages and adaptability and complexity of intervention^(1,9)^. The review of these intervention characteristics allows implementation researchers to assess whether the intervention is ready for scaling-up in larger implementation settings^(10)^. Our conceptual framework proposes four key characteristics of an intervention to make a scaling-up decision: intervention source, evidence strength and quality, relative advantages and adaptability and complexities^(1)^.

When an intervention is identified through authentic sources and the evaluation demonstrates high-quality evidence, then the implementation stakeholders, including implementers, beneficiary groups and funders, may develop confidence in the intervention. The current study focussed on the micronutrient powder (MNP) supplement that was developed and tested efficacy and effectiveness through several randomised controlled trials and corroboration, and such evidence has been published in peer-reviewed journals^(11–13)^. It is generally assumed that the results of a trial published in a peer-reviewed journal constitute authentic evidence, as a peer review process acts to filter out low-quality research, and scholarly research is endorsed by peers who are experts in the same field^(14)^.

The relative advantages of home-fortification intervention have been well established through several effectiveness trials^(11,15–17)^. Compared to other interventions (e.g. Fe syrups and paediatric drops) for childhood anaemia, the MNP home fortification has some advantages, such as MNP sachets being easy to distribute and store by community-level health workers and being easy to use by child caregivers^(11)^. MNP home-fortification with food has fewer side effects compared with other similar interventions, such as supplements in Fe syrups and drops^(15)^. The economic evaluation of MNP home fortification demonstrates that it is cost effective in terms of per capita disability-adjusted life years (fifth article of this supplement)^(8,16,17)^.

To reach the targeted children, multiple models, platforms and channels have identified potential for MNP delivery, including the use of community-based distribution channels^(18,19)^. At the community level, the MNP intervention needs community-based facilities and a dedicated skilled health workforce at the community level, as the intervention needs to be implemented at the household level^(19–21)^. BRAC, a development organisation, based in Bangladesh, aimed to reduce Fe deficiency anaemia (termed as anaemia) by delivering home fortification with MNP among children aged 6–59 months^(7,20)^. A detailed description of BRAC home fortification is available in Paper 4 of this supplement^(7)^. The availability of BRAC community health workers and implementation facilities across sub-districts and districts in Bangladesh are key advantages for BRAC to scale up MNP intervention at the national level^(21,22)^.

## Implementation fidelity: context and process of BRAC home fortification

The second component of the framework is implementation fidelity. In the scaling-up of an intervention, it is important to understand implementation fidelity and measure it in a timely manner^(23)^ to understand whether there is any gap in the implementation which may need to be addressed. Evaluation of implementation fidelity allows implementers to better understand the intervention outcomes and correlate why a certain outcome is observed^(24,25)^. Our framework suggests that evaluators should assess the implementation process and outcomes to understand implementation fidelity and investigate the implementation contexts at the individual, programme and organisation levels^(1,6)^. Timely understanding of the implementation process facilitates endorsement by implementers to adjust implementation gaps through timely course correction that eventually ensures high implementation fidelity and expected intervention outcomes^(1,6)^.

The framework proposed to assess implementation contexts and processes and timely investigate the bottlenecks in implementation fidelity^(1,7)^. We used both qualitative and quantitative analyses to investigate key contextual factors associated with the performance of BRAC community health workers. The performance of BRAC community health workers has been identified as an important issue in the implementation fidelity of BRAC community health workers. Home visits by BRAC community health workers was a key performance indicator for its home-fortification programme^(21)^. The qualitative analysis of BRAC home-fortification programmes published elsewhere^(22)^ identified a range of factors at the individual, community, programme and organisational levels, which influenced the performance of BRAC community health workers with respect to implementing the home-fortification programme^(22)^.

## Real-time course correction of programme implementation plan

As discussed above, with respect to the key findings related to the implementation process and context of the BRAC home-fortification intervention, it was revealed that implementation fidelity critically suffered from several implementation bottlenecks and required the attention of implementers to address them. The fourth article^(5)^ of this supplement documented how BRAC considered these evaluation findings to promptly address the implementation bottlenecks in the home-fortification programme while it was still in operation. This article also documented that timely course correction of the programme’s implementation plan improved the programme’s outcomes. Globally, there are extremely limited examples available of a large-scale programme being implemented at the national level and demonstrating improvement over the period through evidence-based programme course correction.

One of the key components of our implementation science framework is the concurrent evaluation and use of real-time evaluation data to address and overcome any obstacles to implementation^(23)^. The concurrent evaluation data can be used in a way that is consistent with feedback-loop mechanisms to assess a ‘snapshot’ of an individual’s or team’s advancement in institutionalising the intervention in a complex real-world setting^(23,24)^. These findings also provided additional evidence and highlighted the implementation of the intervention over the period, establishing that the transformation was adopted^(23,25)^.

Despite the importance of evaluation in implementation science, there is a recommendation to consider evaluation separate from implementation science^(26)^, which may reduce opportunity for timely assessing the implementation gaps and course correction of these gaps when it is essential. Paper 4 of this supplement described how the BRAC home-fortification programme was difficult to implement at the community level in the initial years due to the low performance of BRAC community health workers. A concurrent evaluation found that several contextual factors associated with the different levels of the BRAC home-fortification programme influenced the programme outcomes. The lessons learned through evaluation supported successful programme implementation and increased the confidence of implementers and investors in the evaluator. This reduced the anxiety usually observed among implementers towards evaluation and positively impacted the relationship between evaluators and implementers^(24)^. A previous paper suggested a feedback loop mechanism, emphasising ongoing follow-up, gap identification, adoption and extended uptake phases, so that with each cycle the intervention becomes more decisively rooted within a system^(23)^.

## Concurrent evaluation improved programme outcomes

A rigorous evaluation strategy is required from the very beginning of implementation science (to identify an effective intervention)^(27)^. Several implementation science frameworks dealing with nutrition interventions suggest an evaluation strategy to measure the implementation context, process, outcome and impacts^(28–30)^. In implementation science, the evaluation should not be only concerned with measuring the implementation outcome or impact but also with considering how an intervention generates certain outcomes: specifically, how evaluation evidence helps to drive implementation improvements^(24,31)^.

The BRAC home-fortification programme benefited from concurrent evaluation, which included several evaluation activities, such as a coverage survey, qualitative investigations, a process evaluation, operations research and an economic evaluation^(5)^. The coverage survey measured the programme’s coverage, identifying barriers to coverage and measuring the impact of the programme. The qualitative investigations were supplemented by coverage survey findings and generated in-depth evidence to support the holistic interpretation of the facts. The process evaluation tracked the implementation process of the home-fortification programme, identified challenges in the implementation process and performed an in-depth investigation to understand the root causes of the challenges. The operations research identified additional interventions that helped to improve the performance of BRAC community health workers. The final round of the coverage survey (i.e. three endline surveys in the three-programme platforms) measured the impact of the programme on anaemia reduction.

## Sustainability of the BRAC home-fortification programme

The issues of sustainability are becoming increasingly important in implementation science and are of interest among researchers, implementers, donors and stakeholders who wish to improve the sustainability of effective public health programmes^(32–35)^. Previous literature suggests considering sustainability in implementation science frameworks as a theory-driven approach to guide the design, development, implementation, evaluation and sustainability of interventions^(36)^. Our conceptual framework for implementation science proposes that a conceptual understanding of sustainability is important and suggests the need to measure the dimensions and determinants of sustainability in a real-world setting. The dimensions related to the sustainability of the BRAC home-fortification programme are dose, reach, fidelity and adaptability, and the determinants are the characteristics of the BRAC home-fortification programme, the organisational factors and the contextual factors^(1)^. The dose refers to the inputs and continuing investments in the home-fortification interventions, such as continuous training for the BRAC community health workers. The reach refers to the coverage of the home-fortification programme, for example, whether the home-fortification intervention maintains a sustained coverage. The fidelity refers to the quality of the implementation and whether the BRAC home-fortification programme maintains implementation quality after the phase-out of external funding. The final dimension is adaptability, which concerns whether the BRAC home fortification is continuously adapting to the implementation contexts and maintaining a high level of implementation fidelity.

This concurrent evaluation did not assess the dimensions of sustainability of the BRAC home-fortification programme. It was designed to assess the sustainability of an externally funded programme after 2 to 3 years of the phase-out of external funding supports^(36–38)^. The BRAC home-fortification funding phased out in December 2018. Now BRAC may initiate assessing the sustainability of its home-fortification programme.

There is much positive evidence available regarding the determinants of the sustainability of the BRAC home-fortification programme. The programme used a market-based strategy, which usually ensures better financial sustainability compared with a subsidised programme, where MNP sachets are distributed free of cost to the caregiver. Another important characteristic of the BRAC programme is the use of volunteer community health workers and the incentivisation of them through several methods, such as allowing them to share in the profit margin of BRAC’s product sales, supporting them to maintain a revolving fund and providing them with guidance for income generation. These incentives motivated the BRAC community health workers to better perform in the programme and assumed that they may continue after the phase-out of funding supports for the programme. Previous literature also suggests that both financial and non-financial incentives motivate community health workers and sustain programme implementation with better outcomes^(39–42)^.

BRAC has a good organisational set-up across Bangladesh. BRAC programmes cover every district in the country with a strong network from the community to districts, which supports community-level services. At the community level, BRAC has well-equipped field offices that can be used to provide programme-specific training for community health workers. BRAC-paid community health workers (e.g. Shasthya Kormi) are skilled service providers who provide a range of health services to communities. BRAC recently introduced a sustainable community health worker model, wherein the community health workers and their supervisor sell their services to the community and generate revenue for BRAC.

## Limitations and strengths

This research project has several limitations and strengths, which have been discussed in the individual article included in this supplement. The conceptual framework was developed based on peer-reviewed articles only and did not include the grey literature, book or book chapters that may have used implementation science frameworks. The review of other sourced frameworks may be worthwhile to identify any new dimensions or components of implementation science that may benefit the comprehensiveness of the conceptual framework. The concurrent evaluation was the main source of data for all the articles included in this supplement, and the evaluation did not consider an appropriate comparison group, which limits our ability to measure counterfactuals of the programme outcomes that we measured. The strength of this research includes the use of multi-methods evaluation data, which allowed us to comprehensively assess programme implementation and outcome. Concurrent evaluation is a flexible strategy; it permits fine-tuning of the evaluation activities during implementation, adjusts with programme needs and eventually improves programme outcomes.

## Conclusions

Overall, the use of a conceptual framework of implementation science framework in the BRAC home-fortification programme was found to be useful. The framework encouraged a comprehensive approach that took account of real-world complexity in implementing the intervention. Considering real-world complexity allowed the implementation researchers to understand the barriers adequately and adjust them before programme outcomes were generated. BRAC community health workers can be instrumental in implementing home fortification with MNP interventions in low-income settings. Timely consideration of potential challenges facing community health workers in home-fortification implementation may help implementers improve the implementation plan and sustain the programme after the phase-out of external funding supports. The lessons learned in this supplement may guide researchers, policymakers and key stakeholders in the nutrition field to scale up new nutrition interventions and sustain them until malnutrition is alleviated. The conceptual framework for implementation science that we have developed and tested retrospectively may need further testing prospectively for a community-based programme implementation.
